# Structure of the *Reston ebolavirus* VP30 C-terminal domain

**DOI:** 10.1107/S2053230X14003811

**Published:** 2014-03-25

**Authors:** Matthew C. Clifton, Robert N. Kirchdoerfer, Kateri Atkins, Jan Abendroth, Amy Raymond, Rena Grice, Steve Barnes, Spencer Moen, Don Lorimer, Thomas E. Edwards, Peter J. Myler, Erica Ollmann Saphire

**Affiliations:** a Seattle Structural Genomics Center for Infectious Disease (SSGCID), 307 Westlake Avenue North, Suite 500, Seattle, WA 98109, USA; b Emerald Bio, Preston Court, Bedford, MA 01730, USA; c Department of Immunology and Microbial Science, The Scripps Research Institute, 10550 North Torrey Pines Road, IMM-21, La Jolla, CA 92037, USA; d Emerald Bio, 7869 NE Day Road West, Bainbridge Isle, WA 98110, USA; e Seattle Biomedical Research Institute, 307 Westlake Avenue North, Suite 500, Seattle, WA 98109, USA; f The Skaggs Institute for Chemical Biology, 10550 North Torrey Pines Road, IMM-21, La Jolla, CA 92037, USA

**Keywords:** ebolaviruses, *Reston ebolavirus*, VP30 C-terminal domain

## Abstract

The ebolaviruses can cause severe hemorrhagic fever. Essential to the ebolavirus life cycle is the protein VP30, which serves as a transcriptional cofactor. Here, the crystal structure of the C-terminal, NP-binding domain of VP30 from *Reston ebolavirus* is presented. Reston VP30 and Ebola VP30 both form homodimers, but the dimeric interfaces are rotated relative to each other, suggesting subtle inherent differences or flexibility in the dimeric interface.

## Abstract 2

The crystal structure of the *Reston ebolavirus* VP30 C-terminal domain shows a rotated interface in comparison to the previous structure of the *Zaire ebolavirus* VP30 C-terminal domain.

## 1. Introduction   

Ebolaviruses can cause hemorrhagic fever in humans, with a fatality rate as high as 90% (Burke *et al.*, 1978 ▶). Four of the five ebolaviruses, including *Ebola virus* (EBOV; formerly *Zaire ebolavirus*), are found in Africa. However, *Reston ebolavirus* (RESTV) is uniquely Asian in origin. RESTV has been identified in bats and primates (Miranda *et al.*, 1999 ▶; Rollin *et al.*, 1999 ▶; Taniguchi *et al.*, 2011 ▶), as well as swine (Barrette *et al.*, 2009 ▶; Sayama *et al.*, 2012 ▶). However, in humans RESTV appears to be nonpathogenic and transmits poorly for reasons that are not fully understood (Miranda *et al.*, 1999 ▶).

Four proteins are essential for viral transcription: nucleoprotein (NP), VP30, VP35 and the RNA-dependent RNA polymerase (L) (Mühlberger *et al.*, 1999 ▶). VP30 appears unique to the filoviruses and is essential for rescuing recombinant ebolavirus (Enterlein *et al.*, 2006 ▶; Theriault *et al.*, 2004 ▶). VP30 allows the polymerase to read beyond a *cis*-RNA element in the NP mRNA 5′ untranslated region (Weik *et al.*, 2002 ▶) and to re-initiate transcription at gene junctions (Martínez *et al.*, 2008 ▶). Furthermore, VP30 phosphorylation modulates the composition and function of the RNA synthesis machinery (Biedenkopf *et al.*, 2013 ▶; Martinez *et al.*, 2011 ▶).

Both VP30 N- and C-terminal domains have been associated with oligomerization and nucleocapsid-association functions (Hartlieb *et al.*, 2003 ▶, 2007 ▶). A basic cluster in the C-terminal domain (CTD) contributes to the association of VP30 with NP and is essential for transcription activation (Hartlieb *et al.*, 2007 ▶). Previous crystallo­graphic studies demonstrated that the EBOV VP30 CTD forms a dimer by donation of the C-terminal helix 7 to the neighboring monomer (Hartlieb *et al.*, 2007 ▶). To date, the EBOV VP30 CTD is the only filovirus VP30 structure available. Here, we present the crystal structure of the VP30 CTD from *Reston ebolavirus*.

## 2. Materials and methods   

### 2.1. Cloning, expression and protein purification   

RESTV (strain Reston-89) VP30 CTD, residues 142–266, was cloned using polymerase incomplete primer extension (PIPE; Klock & Lesley, 2009 ▶) into a modified *Escherichia coli* pET28 vector system engineered for N-terminal hexahistidine-Smt tags (clone ID EbreA.17250.a.D11). Expression was performed in *E. coli* BL21 (DE3) cells in TB medium (Teknova) at 37°C with 50 µg ml^−1^ kanamycin. Cells were induced at an OD_600_ of 0.7 with 1 m*M* IPTG and incubated overnight at 25°C.

**Figure 1:**
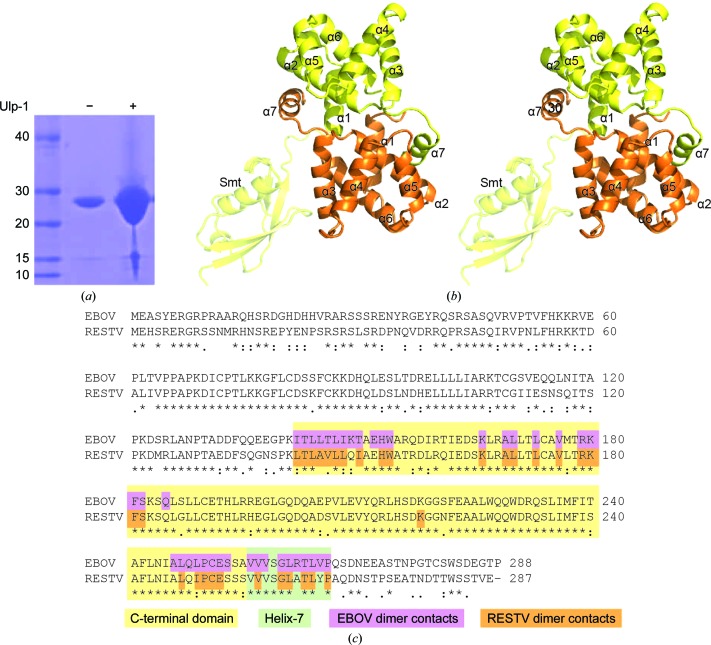
Overall *Reston ebolavirus* VP30 C-terminal domain structure. (*a*) Attempted cleavage of Smt-VP30 CTD with Ulp-1 protease. Lane 1, protein marker (labeled in kDa); lane 2, undigested protein; lane 3, Ulp-1 digestion. (*b*) The VP30 monomers forming the dimer are shown in orange and yellow. The ordered Smt domain is attached to the yellow VP30. The image is shown in wall-eyed stereo. (*c*) Sequence alignment of VP30 highlighting amino acids participating in the dimer interface. Residues in the dimer interfaces where amino acids are within 4 Å of the other chain in the corresponding structures are highlighted in purple (EBOV) and orange (RESTV).

Cells were resuspended in lysis buffer [25 m*M* Tris–HCl pH 8.0, 200 m*M* NaCl, 50 m*M* arginine, 1 m*M* tris(2-carboxyethyl)phosphine (TCEP), 10 m*M* imidazole, 0.5% glycerol, 125 U Benzonase, 0.71 mg ml^−1^ lysozyme and one EDTA-free complete protease inhibitor tablet (Roche)] and lysed *via* sonication. The lysate was clarified by centrifugation and filtration. The protein was eluted from HiTrap Ni^2+^ Chelating columns (GE Healthcare) with an imidazole step gradient. Pooled fractions were incubated for 18 h with Ulp-specific protease 1 (Ulp-1), intended to cleave the Smt domain from VP30. However, SDS–PAGE analysis showed that the His-Smt tag was unable to be removed (Fig. 1 ▶ *a*). The protein was dialyzed against 25 m*M* Tris pH 8.0, 200 m*M* NaCl, 1 m*M* TCEP, 1% glycerol for 12 h and was concentrated to 18 mg ml^−1^ for crystallization. Macromolecule-production information is given in Table 1 ▶.

### 2.2. Crystallization   

Crystals grew by vapor diffusion in sitting-drop trays in 10% PEG 6000, 100 m*M* HEPES pH 7.0 in 2–3 weeks using 0.4 µl protein solution and 0.4 µl reservoir solution at 16°C. The rod-shaped crystals were cryoprotected with 20% ethylene glycol prior to flash-cooling in liquid nitrogen. Details are given in Table 2 ▶.

### 2.3. Data collection and processing   

Data were collected on beamline 5.0.1 at the Advanced Light Source with the detector set at a distance of 300 mm, with 0.5° oscillations and 5 s exposures for a total of 300 frames The data were reduced using *XDS* (Kabsch, 2010 ▶) and *XSCALE*. Data-collection and processing statistics are given in Table 3 ▶.

### 2.4. Structure solution, refinement and analysis   

The structure was determined by molecular replacement using *Phaser* (McCoy *et al.*, 2007 ▶) with the structure of EBOV VP30 CTD in monomeric form (Hartlieb *et al.*, 2007 ▶; PDB entry 2i8b) as a search model. The structure was refined with *REFMAC* (Murshudov *et al.*, 2011 ▶) and model building was performed with *Coot* (Emsley & Cowtan, 2004 ▶). The final structure was validated with *MolProbity* (Chen *et al.*, 2010 ▶). Protein interfaces were analyzed using *PISA* (Krissinel & Henrick, 2007 ▶) and the shape-correlation statistic S_C_ (Lawrence & Colman, 1993 ▶).

## 3. Results and discussion   

In the crystal structure, there are two copies of the RESTV VP30 CTD in the asymmetric unit (Fig. 1 ▶ *b*). The VP30 domains retain their Smt fusion domains from purification (Fig. 1 ▶ *a*). One of the Smt domains helps facilitate crystal-packing interactions, whereas the second Smt domain is disordered. The two copies of RESTV VP30 in the asymmetric unit are nearly identical in structure and superimpose with an r.m.s.d. of 0.76 Å.

The fold of RESTV VP30 CTD is helical and closely resembles that of EBOV VP30, with an r.m.s.d. of 1.15–2.14 Å separating the C^α^ backbones of the EBOV and RESTV structures. Like EBOV VP30 (Hartlieb *et al.*, 2007 ▶), RESTV VP30 forms a dimer by packing of its extended C-terminal helix 7 into a conserved hydrophobic face on the neighboring monomer (Fig. 1 ▶ *c*). Dimerization in both EBOV and RESTV VP30 is also facilitated by similar hydrophobic interactions between the globular domains and a conserved hydrogen bond between the side chain of Lys180 in helix 2 and the main chain of Cys251 in the linker between helices 6 and 7.

**Figure 2:**
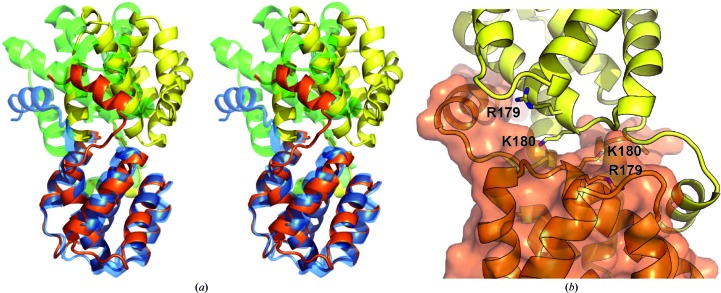
Reston VP30 CTD dimer. (*a*) Superimposition of the EBOV and RESTV VP30 C-terminal domain structures using a single domain shows a rotation about the dimer interface. The RESTV VP30 molecules are colored orange and yellow, while the EBOV VP30 molecules are colored blue and green. For clarity, the Smt is not illustrated. (*b*) The altered orientation of the RESTV VP30 CTD buries the basic residues Arg179 and Lys180 in the dimer interface.

Superposition of the RESTV and EBOV VP30 CTD structures shows that the RESTV VP30 CTD assembly is significantly rotated about the dimer interface compared with EBOV (Fig. 2 ▶ *a*). The domain rotation lowers the buried surface area on each monomer (RESTV, 1630 Å^2^; EBOV, 1900 Å^2^) but maintains a similar surface complementarity (RESTV, S_C_ = 0.69; EBOV, S_C_ = 0.68), suggesting that the RESTV VP30 CTD dimer is a relevant conformation (Lawrence & Colman, 1993 ▶), and conservation of residues within the dimer interface suggests this conformation could also exist for EBOV.

It is difficult to discern the impact of the crystal-packing inter­actions on the EBOV or RESTV VP30 CTD conformations, as neither conformation is compatible with the packing of the other ebolavirus species. Additionally, the Smt domain in the RESTV VP30 CTD structure contributes to the crystal packing as well as making contacts with the opposite chain in the asymmetric unit (buries 545 Å^2^). However, there is no apparent structural reason for the Smt domain to constrain the VP30 conformation and the Smt domain and VP30 CTD are not expected to form a stable complex in solution (Krissinel & Henrick, 2007 ▶).

The observed rotation in the RESTV VP30 dimer buries Arg179 and Lys180 (Fig. 2 ▶ *b*), which are instead solvent-exposed in the EBOV structure. These residues are important in VP30 function and their mutation is detrimental to VP30 oligomerization, NP binding and transcription initiation (Hartlieb *et al.*, 2007 ▶). The occlusion of Arg179 and Lys180 in the RESTV dimer interface suggests that mutations at these positions could disrupt VP30 dimerization.

EBOV and RESTV VP30 have 84% sequence identity within the CTD and conserve both the overall structure and hydrophobic interfaces (Fig. 1 ▶ *c*). While we are unable to rule out the influence of the Smt on the conformation of the RESTV dimer, the observation of two dimer conformations in the available structures suggests that there may be inherent differences between the African and Asian viral species. Alternatively, the VP30 CTD could adopt multiple conformations in solution: structural changes in VP30 may reflect its role in modulation of RNA synthesis or another role in the ebolavirus lifecycle.

## Supplementary Material

PDB reference: *Reston ebolavirus* VP30 C-terminal domain, 3v7o

## Figures and Tables

**Table 1:** Macromolecule production

Source organism	*Reston ebolavirus* strain Reston-89
DNA source	Synthetic
Forward primer	AAC AAA TCG GTG GAC TCA CTC TGG CAG TGT TAC TGC AGA T
Reverse primer	GGC CGC AAG CTT TTA CGT GCT GTT ATC CTG AGC AGG GTA C
Cloning vector	pET-28a
Expression vector	pET-28a
Expression host	*E. coli* BL21 (DE3)
Complete amino-acid sequence of construct produced	MGHHHHHHSGEVKPEVKPETHINLKVSDGSSEIFFKIKKTTPLRRLMEAFAKRQGKEMDSLRFLYDGIRIQADQTPEDLDMEDNDIIEAHREQIGGLTLAVLLQIAEHWATRDLRQIEDSKLRALLTLCAVLTRKFSKSQLGLLCETHLRHEGLGQDQADSVLEVYQRLHSDKGGNFEAALWQQWDRQSLIMFISAFLNIALQIPCESSSVVVSGLATLYPAQDNST

**Table 2:** Crystallization

Method	Vapor diffusion, sitting drop
Plate type	96-well Compact Jr plates (Emerald Bio)
Temperature (°C)	16
Protein concentration (mg ml^−1^)	18
Buffer composition of protein solution	25 m*M* Tris pH 8.0, 200 m*M* NaCl, 1 m*M* TCEP, 1% glycerol
Composition of reservoir solution	10% PEG 6000, 100 m*M* HEPES pH 7.0
Volume and ratio of drop	0.8 µl, 1:1
Volume of reservoir (µl)	100

**Table 3:** Data-collection and processing statistics Values in parentheses are for the highest shell.

Data collection and processing	
Diffraction source	ALS 5.0.1
Wavelength (Å)	0.97740
Temperature (°C)	−173
Detector	ADSC Quantum 210 CCD
Crystal-to-detector distance (mm)	300
Rotation range per image (°)	0.5
Total rotation range (°)	120
Exposure time per image (s)	5
Space group	*P*2_1_2_1_2_1_
Unit-cell parameters (Å, °)	*a* = 49.3, *b* = 93.7, *c* = 111.2, α = β = γ = 90
Mosaicity (°)	0.13
Resolution (Å)	50–2.25 (2.31–2.25)
Total reflections	120225 (8910)
Unique reflections	25173 (1831)
Completeness (%)	99.7 (100.0)
Multiplicity	4.8 (4.9)
〈*I*/σ(*I*)〉	15.2 (3.2)
*R*_p.i.m._	0.048 (0.35)
*R*_merge_	0.08 (0.51)
Overall *B* factor from Wilson plot (Å^2^)	32.8
Refinement	
Resolution range (Å)	50–2.25 (2.31–2.25)
Completeness (%)	99.7 (100)
No. of reflections, working set	23841 (1577)
No. of reflections, test set	1280 (95)
Final *R*_cryst_ (%)	0.19 (0.24)
Final *R*_free_(%)	0.23 (0.32)
Cruickshank DPI (Å)	0.20
No. of non-H atoms	
Protein	2554
Ligand	8
Water	150
Total	2712
R.m.s. deviations	
Bonds (Å)	0.011
Angles (°)	1.33
Mean *B* factors (Å^2^)	
Overall	32.6
Protein	32.4
Ligand	44.0
Water	34.9
Model statistics	
Ramachandran plot	
Favored regions (%)	98.8
Additionally allowed (%)	0.9
*MolProbity* score	1.23 [100th percentile]
Clashscore	1.95 [100th percentile]
PDB code	3v7o
